# Partitioning variance in reproductive success, within years and across lifetimes

**DOI:** 10.1002/ece3.10647

**Published:** 2023-11-20

**Authors:** Robin S. Waples

**Affiliations:** ^1^ Northwest Fisheries Science Center National Marine Fisheries Service, National Oceanic and Atmospheric Administration Seattle Washington USA

**Keywords:** computer simulations, effective population size, opportunity for selection, population genetics, reproductive skew, variance components

## Abstract

Variance in reproductive success ($s_{k}^{2}$, with *k* = number of offspring) plays a large role in determining the rate of genetic drift and the scope within which selection acts. Various frameworks have been proposed to parse factors that contribute to $s_{k}^{2}$, but none has focused on age‐specific values of $\phi =s_{k}^{2}/\bar{k}$, which indicate the degree to which reproductive skew is overdispersed (compared to the random Poisson expectation) among individuals of the same age and sex. Instead, within‐age effects are generally lumped with residual variance and treated as “noise.” Here, an ANOVA sums‐of‐squares framework is used to partition variance in annual and lifetime reproductive success into between‐group and within‐group components. For annual reproduction, the between‐age effect depends on age‐specific fecundity (*b*_*x*_), but relatively few empirical data are available on the within‐age effect, which depends on *ϕ*_*x*_. By defining groups by age‐at‐death rather than age, the same ANOVA framework can be used to partition variance in lifetime reproductive success (LRS) into between‐group and within‐group components. Analytical methods are used to develop null‐model expectations for random contributions to within‐group and between‐group components. For analysis of LRS, random variation in longevity appears as part of the between‐group variance, and effects (if any) of skip breeding and persistent individual differences contribute to the within‐group variance. Simulations are used to show that the methods for variance partitioning are asymptotically unbiased. Practical application is illustrated with empirical data for annual reproduction in American black bears and lifetime reproduction in Dutch great tits. Results show that overdispersed within‐age variance (1) dominates annual $s_{k}^{2}$ in both male and female black bears, (2) is the primary factor that reduces annual effective size to a fraction of the number of adults, and (3) represents most of the opportunity for selection. In contrast, about a quarter of the variance in LRS in great tits can be attributed to random variation in longevity, and most of the rest is due to modest differences in fecundity with age estimated for a single cohort of females. R code is provided that reads generic input files for annual and lifetime reproductive success and allows users to conduct variance partitioning with their own data.

## Graphical abstract

An ANOVA framework is used to partition variance in reproductive success into within‐group and between‐group components. Worked examples illustrate how this variance partitioning provides new insights into factors that reduce the effective size: census size ratio and that provide the greatest opportunities for natural selection to act upon.

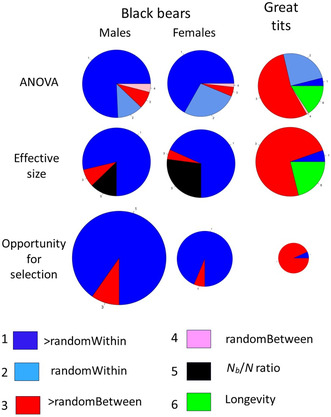

## 1 INTRODUCTION

Variation among individuals is the stuff of evolution. Evolution by natural selection requires individual variation in heritable phenotypic traits affecting fitness. Over a century ago, RA Fisher (1918) introduced the term “variance” (the square of the standard deviation) as the preferred metric for measuring this variation, and immediately efforts began to identify different factors contributing to an overall variance. Perhaps the most famous such example was by Lewontin (1972), who estimated that only about 15% of the total molecular genetic variance in humans was due to differences between races or between populations within races, with the remaining 85% found among individuals within populations. The amount of data Lewontin had available at the time was extremely limited by today's standards, but his qualitative conclusions have proved surprisingly robust and influential across a half‐century (Novembre, 2022).

Another kind of variance—in reproductive success, measured as the number of offspring, *k*, contributed to the next generation—is also crucially important to evolution. The ratio of variance‐to‐mean offspring number $\left(\phi =\sigma_{k}^{2}/\bar{k}\right)$ has been termed the “Index of Variability” (Crow & Morton, 1955), and this index is the primary factor that determines to what extent (if any) the effective population size (*N*_*e*_) is less than the census size (*N*) (Caballero, 1994; Crow & Denniston, 1988). A related index defined by Crow (1958); $I=\sigma_{k}^{2}/{\bar{k}}^{2}=$ the variance in relative fitness) has come to be known as the opportunity for selection because its sets an upper limit to the rate of evolutionary adaptation. Variance in offspring number appears in the numerator of both of these indices and consequently has as large role in determining both the rate of genetic drift and the scope within which selection can act.

The major goal of this paper is to develop an approach analogous to Lewontin's, but instead of apportioning genetic data based on race or geography, we will be concerned with partitioning the overall variance in reproductive success into within‐age and between‐age components. Emphasis is on the large fraction of the world's species that are age structured and iteroparous, with overlapping generations and strongly seasonal (birth‐pulse) reproduction (Caswell, 2001). For these species, it is important to consider two different frameworks for measuring reproductive success: within seasons or time periods (hereafter assumed to be years), and over lifetimes (quantified as lifetime reproductive success, or LRS; aka lifetime reproductive output, van Daalen & Caswell, 2017). For annual reproduction, two systematic components contribute to the overall variance in offspring number: a between‐age effect and a within‐age effect. The between‐age effect depends on how expected reproductive success varies with age, as reflected in age‐specific expected fecundity values (*b*_*x*_) from a life table. The within‐age effect depends on age‐specific values of *ϕ*_*x*_, which unfortunately are rarely reported in the literature. As a consequence, how *ϕ*_*x*_ varies across species and between ages within species is largely unknown.

For lifetime reproduction, the relevant metric is variance in LRS, $\sigma_{k•}^{2}$ (Brown, 1988; Hill, 1972; Tuljapurkar et al., 2020). Lifetime $\sigma_{k•}^{2}$ is affected by age‐specific variation in *b*_*x*_ and *ϕ*_*x*_, as well as another factor: longevity. All else being equal, individuals that live longer have more opportunities to reproduce, which increases disparity in lifetime offspring number between individuals and increases $\sigma_{k•}^{2}$. Caswell (2001) and van Daalen and Caswell (2017) use the term “Markov chains with rewards” to describe the random aspects of this process of accumulating LRS, and variation in longevity can be the dominant factor contributing to variance in LRS (e.g., Newton, 1989).

The number of components of reproductive success that potentially could be identified is essentially unlimited, and a wide variety of frameworks for doing this have been proposed over the years (Arnold & Wade, 1984; Broekman et al., 2020; Brown, 1988; Ferguson & Fairbairn, 2001; van Noordwijk & van Balen, 1988). Some researchers have paid attention to within‐age contributions to reproductive variance, but if so it has generally been to identify ages for which this variance is relatively large (e.g., the Siberian jay example in Engen et al., 2010; the moose example considered by Lee et al., 2020; and the sagebrush case study by Snyder et al., 2021). In linear modeling, the within‐age component typically is incorporated into the residual or error variance and treated as noise.

All of these approaches can provide useful insights, depending on one's objectives and the kinds of data that are available, but none has focused on quantifying the overall contribution from variance in offspring number among individuals of the same age and sex. This is an important data gap; the residual variance often dominates the overall variance and, as shown in the black bear example below, the degree to which reproductive variance is overdispersed can vary by age and sex. Important insights into the potential for selection to act and the factors responsible for reducing *N*_*e*_ compared to *N* can be missed when the within‐age variances are largely ignored.

In what follows, I first describe a simple one‐way ANOVA framework within which researchers can partition variance in annual reproductive success into within‐age and between‐age effects and can partition variance in LRS into within‐age, between‐age, and longevity effects. Analytical methods are also developed that allow researchers to calculate what fraction of these variance components can be attributed to random (Poisson) stochasticity in reproduction and survival. Modern molecular tools and improved methods for parentage analysis make it easier than ever to collect empirical data on variance in offspring number. Analysis of annual data is important because any long‐term study begins with data collected for individual years, and many empirical studies never encompass entire lifespans for the focal species (Nishida, 1989). Furthermore, the distribution of annual reproductive success provides key insights into mating systems, and the most direct way to study sexual selection is by collecting data on all mature individuals that co‐occur at the same time and place. That is not the case when analyzing only LRS data. Understanding the relative magnitude of between‐age and within‐age effects should lead to richer insights into mating systems and reproductive biology, as well as an increased ability to predict evolutionary responses to environmental changes that can affect fitness.

## 2 METHODS

See Table 1 for notation and definitions of terms.

**TABLE 1:** Notation.

*x*	Age (in years)
α	Age at maturity
ω	Maximum age
*q*	Age at death
*n*	Number of adult age classes = number of groups in ANOVA
*v*_*x*_	Probability of surviving from age *x* to age *x* + 1
*l*_*x*_	Cumulative probability of surviving to age *x*, with *l*_*1*_ = 1
*N*_1_	Cohort size = number of offspring of one sex produced per time period that survive to age 1
*N*_*x*_	Number of individuals of age *x* alive at any given time; *N*_*x*_ = *N*_1_ *l*_*x*_
*D*_*q*_	Number of individuals that died after reaching age *q*; *D*_*q*_ = *N*_*q*_–*N*_*q +* 1_
*N*_*T*_	Total number of individuals of all ages alive at any given time; *N*_*T*_ = Σ*N*_*x*_
*N*_*A*_	Number of adults with age ≥ *α* alive at any given time
*Q*	An index of sampling intensity relative to sampling all offspring in a stable population
*b*_*x*_	Expected number of offspring in one time period for parents of age *x*
$k_{i,x}$	Actual number of offspring produced in one time period by parent *i* of age *x*
${\bar{k}}_{x}$	Actual mean *k* for parents of age *x*
${\bar{k}}_{∙}$	Actual mean *k* for all parents
$\bar{b}$	Parametric mean offspring number for all parents in one time period
${\hat{b}}_{x}$	An estimate of *b*_*x*_ obtained by rescaling ${\bar{k}}_{x}$ by sampling intensity; ${\hat{b}}_{x}={\bar{k}}_{x}/Q$
$\sigma_{k}^{2}$	Parametric variance of *k* for all parents
$\sigma_{k_{x}}^{2}$	Parametric variance of *k* for adults of age *x*
*ϕ*_*x*_	Ratio of parametric variance to mean reproductive success in one time period for adults of age *x*; *ϕ*_*x*_ = $\sigma_{k_{x}}^{2}$ */b*_*x*_
$s_{k_{x}}^{2}$	Unbiased sample variance of *k* for adults of age *x*
${\hat{\sigma }}_{k_{x}}^{2}$	An estimate of $\sigma_{k_{x}}^{2}$ obtained be rescaling $s_{k_{x}}^{2}$ according to Equations 7a and 7b
$k{•}_{i,q}$	Total lifetime number of offspring produced by individual *i* that died at age *q*
$k{•}_{q}$	Mean $k{•}_{i,q}$ for all individuals that died at age *q*
$s_{k_{x}}^{2}$	Unbiased sample variance of $k{•}_{i,q}$ for all individuals that died at age *q*
$k{•}_{∙}$	Mean $k{•}_{i}$ for all individuals
$\sigma_{k•}^{2}$	Parametric variance in $k{•}_{i}$ among all *N*_1_ individuals in a cohort
SSB (SSB•)	Between‐age sum of squares for annual (lifetime) reproduction
SSE (SSE•)	Error or within‐age sum of squares for annual (lifetime) reproduction
SST (SST•)	Total sum of squares for annual (lifetime) reproduction
*ρ*_*α,α+*_	Correlation across individuals between the number of offspring produced at the age at maturity (*α*) and the total offspring produced during the rest of their lifetimes.

### 2.1 Demographic model

The focal population is isolated and iteroparous, with separate sexes. Analogous methods apply to both males and females; for simplicity data are considered for a single sex, nominally female. Reproduction follows the discrete‐time, birth‐pulse model (Caswell, 2001), with age indexed by *x*. At age *x*, each individual produces on average *b*_*x*_ offspring and survives to age *x* + 1 with probability *v*_*x*_. Age at maturity (first age with *b*_*x*_ > 0) occurs at age α and maximum age is *ω*. Newborns (age 0) do not reproduce, so *b*_*x*_ is scaled to production of offspring that survive to age 1, when they can be enumerated. Cumulative survival through age *x* is $l_{x}=\prod_{i=2}^{x}v_{i-1}$, with *l*_1_ = 1. In a constant population with each birth cohort consisting of *N*_1_ yearlings, the expected number of individuals of age *x* alive at any given time is *E*(*N*_*x*_) = *N*_1_ *l*_*x*_, and expected total census size is *E*(*N*_*T*_) = ∑*E*(*N*_*x*_) = *N*_1_ *Σ l*_*x*_. Because we are concerned with reproductive success, focus is on the adult population size, $N_{A}=\sum_{x=\alpha }^{\omega }N_{x}$.

Standard life tables provide values for age‐specific survival and fecundity, to which we add a third age‐specific vital rate, $\sigma_{k,x}^{2}$, which is the variance in offspring number (*k*) around the mean for individuals of age *x* (*b*_*x*_). In many cases, it is convenient to deal with the parameter $\phi_{x}=\sigma_{k,x}^{2}/b_{x}$, which is the age‐specific ratio of variance to mean offspring number.

### 2.2 Variance partitioning

Two major goals of this paper are to (1) quantify the relative importance of within‐age and between‐age effects to the overall variance in reproductive success and (2) show how these differences depend on, and can be predicted from, age‐specific vital rates. This is done using a one‐way ANOVA sums‐of‐squares framework, which partitions sources of variation into three components (SST, total sum of squares of deviations; SSB, between‐group sum of squares; and SSE, error or within‐group sums of squares). The sums of squares are additive, such that SST = SSB + SSE, and the groups are the ages in an adult lifespan. In Model II (random effects) ANOVA, this partitioning commonly is done in a “variance components” analysis, which directly estimates the within‐ and between‐group variances that are associated with SSB and SSE. That approach is not used here, for two major reasons. First, variance‐component estimation is done in a random‐effects framework, whereas age is best treated as a fixed effect (Whitlock & Schluter, 2014). Second, although variance components analysis is fairly straightforward in a balanced ANOVA, group sizes in a stable population (*N*_*x*_ = numbers of adults in each age class) generally decline with age. Various methods have been proposed to deal with variance component estimation in unbalanced ANOVA designs, but all have disadvantages and no consensus has emerged regarding the optimal approach (reviewed by Searle (1995).

Instead, the approach used here derives from the fact that the overall parametric variance in offspring number is $\sigma_{k}^{2}=\mathrm{SST}/N_{A}$, so the parametric variance partitioning becomes $\sigma_{k,\text{Within}}^{2}$ = SSE/*N*_*A*_ and $\sigma_{k,\text{Between}}^{2}$ = SSB/*N*_*A*_, such that $\sigma_{k,\text{Within}}^{2}+\sigma_{k,\text{Between}}^{2}=\sigma_{k}^{2}$. It follows that if parametric SSE and SSB can be estimated as a function of the population's vital rates and the experimental design (including sampling intensity), it provides a means for estimating the parametric variance components. Below, this framework is used for analysis of annual and lifetime reproductive success. For simplicity in what follows it is assumed that *α* = 1, but only minor changes to notation are required if age at maturity is >1. If *α* is probabilistic rather than fixed, age‐specific vital rates should reflect overall means and variances across mature and immature individuals (Waples & Reed, 2023).

#### 2.2.1 Annual reproduction

Using the notation described above, the sums‐of‐squares components for annual reproduction are

(1a) $$\mathrm{SSE}=\sum_{x=1}^{n}\sum_{i=1}^{N_{x}}{\left(k_{i,x}-{\bar{k}}_{x}\right)}^{2}$$

(1b) $$\mathrm{SSB}=\sum_{x=1}^{n}N_{x}{\left({\bar{k}}_{x}-{\bar{k}}_{∙}\right)}^{2}$$

(1c) $$\mathrm{SST}=\sum_{i=1}^{N_{x}}\sum_{x=1}^{n}{\left(k_{i,x}-{\bar{k}}_{∙}\right)}^{2}$$

where *n* is the number of adult age classes, $k_{i,x}$ is the number of offspring produced by parent *i* of age *x*, *N*_*x*_ is the group size for age class *x*, and ${\bar{k}}_{x}$ and ${\bar{k}}_{∙}$ are group and overall mean offspring numbers, respectively.

#### 2.2.2 Lifetime reproductive success

In the lifetime reproductive success (LRS) version of the ANOVA framework, individuals are grouped by age‐at‐death (*q*) rather than age, and the symbol • is used to designate lifetime variables. For LRS, the sums of squares become

(2a) $$\text{Within}\;\mathrm{age}-\mathrm{at}-\text{death}:\mathrm{SSE}•=\sum_{q=1}^{n}\sum_{i=1}^{D_{q}}{\left(k{•}_{i,q}-{\bar{k•}}_{q}\right)}^{2},$$

(2b) $$\text{Between}\;\mathrm{age}-\mathrm{at}-\text{death}:\mathrm{SSB}•=\sum_{q=1}^{n}D_{q}{\left({\bar{k•}}_{q}-{\bar{k•}}_{∙}\right)}^{2},$$

(2c) $$\text{Across}\;\mathrm{all}\;\text{individuals}:\mathrm{SST}•=\sum_{q=1}^{n}\sum_{i=1}^{D_{q}}{\left(k{•}_{i,q}-{\bar{k•}}_{∙}\right)}^{2},$$

where *D*_*q*_ is the number of individuals that died after reproducing at age *q* but before reaching age *q* + 1, *k*•_*i,q*_ is the total lifetime number of offspring produced by individual *i* that died at age *q*, ${\bar{k•}}_{q}$ is the mean LRS for individuals that die at age *q*, and ${\bar{k•}}_{∙}$ is mean LRS across all *N*_1_ individuals in a cohort. As with annual reproduction, SST• = SSB• + SSE•.

### 2.3 Effective population size

Mean and variance in offspring number are the major factors that determine effective size. For seasonal reproduction in an age‐structured species, the most relevant effective‐size metric is the effective number of breeders per year (*N*_*b*_) because it relates directly to the annual number of adults (*N*_*A*_). For each sex, *N*_*b*_ can be calculated using the standard discrete‐generation formula for inbreeding effective size (Caballero, 1994; Crow & Denniston, 1988):

(3) Nb=k¯∙NA−1k¯∙−1+σk2k¯∙.

*N*_*b*_ can be calculated separately for males and females and an overall *N*_*b*_ obtained using Wright's (1938) sex‐ratio adjustment. The expected annual variance in offspring number can be calculated from age‐specific vital rates using the definition of a variance: $\sigma_{k}^{2}=E\left(k^{2}\right)-{\left[E\left(k\right)\right]}^{2}.$ Substituting for $E\left(k\right)=\bar{k}$ and $E\left(k^{2}\right)=\Sigma k_{i}^{2}/N$ and rearranging produces an expression for the sum of squared numbers of offspring:

(4) $$\Sigma k_{i}^{2}=N\left(\sigma_{k}^{2}+{\bar{k}}^{2}\right).$$

Equation 4 can applied sequentially to each age and the overall total then used to calculate the overall variance. The black bear example below illustrates these calculations and shows how to separate the within‐age and between‐age contributions to reduction of the key ratio *N*_*b*_/*N*_*A*_.

*N*_*b*_ relates directly to all adults breeding in a single year, but most evolutionary theory depends on effective size per generation (*N*_*e*_). Calculating *N*_*e*_ in iteroparous species is complicated by the necessity of integrating information across multiple episodes of reproduction. Hill (1972) provided the most general solution:

(5) $$N_{e}\approx \frac{4N_{1}T}{{\sigma_{k}^{2}}_{•}+2},$$

where *T* is generation length and ${\sigma_{k}^{2}}_{•}$ is calculated across the *N*_1_ individuals in a cohort.

### 2.4 Opportunity for selection

Application of Crow's *I* has been controversial, in part because it is a function of mean offspring number and hence depends on experimental design and sampling intensity as well as population parameters. The $\Delta_{I}$ metric suggested by Waples (2020) removes the dependency on mean fitness by subtracting the expected contribution to *I* arising from random (Poisson) variance in reproductive success:

(6) $$\Delta_{I}=I-1/{\bar{k}}_{∙}$$

This metric is suitable for use with discrete generations, or for analysis of annual reproductive success in age‐structured species.

For lifetime reproductive success, the inverse of mean LRS accounts for random reproductive success within years and ages. In addition, there is a contribution to lifetime *I*• from random variation in longevity, so the $\Delta_{I}$ metric relevant to LRS is (Waples & Reed, 2023)

$$\Delta_{I•}=I•-\frac{1}{{\bar{k•}}_{∙}}-C,$$

with calculation of *C* as described below.

### 2.5 Sampling designs

For both annual and lifetime reproduction, two sampling designs were considered. In Case 1 (comprehensive sampling), all offspring from a stable population are sampled and assigned to parents. This is the most straightforward design to evaluate because results for empirical data can be related directly to analytical expectation based on parametric vital rates. For annual reproduction in a species with an even primary sex ratio, a stable population of *N*_*A*_ adults produces 2*N*_1_ offspring each year (*N*_1_ of each sex), so under comprehensive sampling the overall mean offspring number is ${\bar{k}}_{∙}=2N_{1}/N_{A}$. For lifetime reproduction, a stable population occurs when the *N*_1_ individuals in a single‐sex cohort each produce an average of two offspring over their lifetime, which again means that a total of 2*N*_1_ offspring are sampled under Case 1. The difference is that for annual reproduction, the offspring are assigned to $N_{A}=\sum_{x=1}^{n}N_{x}$ potential parents while the number of potential parents in each LRS cohort is only *N*_1_, so mean offspring number per parent for annual reproduction is a fraction of what it is for LRS.

Case 2 (generalized sampling) includes Case 1 as a special case but allows for a variety of different experimental designs and a range of sampling intensities. Often only a subset of offspring are sampled, leading to *N*_Offspring_ < 2*N*_1_. Conversely, if young juveniles are sampled in a highly fecund species, *N*_Offspring_ can be >>2*N*_1_. Divergence of *N*_Offspring_ from the stable‐population expectation leads to variation in realized mean offspring number. The ratio *Q* = *N*_Offspring_/(2*N*_1_) is a useful index of sampling intensity, with *Q* = 1 replicating Case 1 (comprehensive sampling). It follows that for annual reproduction *E*(${\bar{k}}_{x}$) = *Qb*_*x*_. Variation in sampling intensity adds complexity to analysis of empirical data because, except in the special case of a Poisson distribution, the variance in offspring number is positively correlated with the mean (Crow & Morton, 1955; Waples, 2002, 2020). This in turn complicates comparisons across populations and years, and even between males and females when the sex ratio is uneven.

If *Q* ≠ 1, it is desirable to standardize the analyses by estimating the expected variance in offspring number when the mean is the value that will produce a stable population (as suggested by Crow & Morton, 1955 and many others). Using our notation for annual reproduction, the rescaling can be done using an age‐specific version of the method of Crow and Morton (1955), as modified by Waples (2002):

(7a) $$s_{k_{x,\text{scaled}}}^{2}={\hat{\sigma }}_{k_{x}}^{2}=b_{x}\left[1+\frac{b_{x}}{{\bar{k}}_{x}}\left(\frac{s_{k_{x}}^{2}}{{\bar{k}}_{x}}-1\right)\right].$$

The unbiased sample variance for age *x* in the raw data is $s_{k_{x}}^{2}=\sum_{i=1}^{N_{x}}{\left(k_{i,x}-{\bar{k}}_{x}\right)}^{2}/\left(N_{x}-1\right)$, and ${\hat{\sigma }}_{k_{x}}^{2}$ is a rescaled version that represents what the variance would be expected to be if sampling had been at the level that would produce mean offspring number $=b_{x}$, instead of the actual raw mean $={\bar{k}}_{x}$. Hence, ${\hat{\sigma }}_{k_{x}}^{2}$ is an estimate of the parametric age‐specific variance in offspring number, obtained by rescaling the raw data. Since $E\left(\frac{b_{x}}{{\bar{k}}_{x}}\right)=1/Q$ and $s_{k_{x}}^{2}/{\bar{k}}_{x}=\phi_{x,\mathrm{raw}}$, Equation 7a can be written as

(7b) $$s_{k_{x,\text{scaled}}}^{2}={\hat{\sigma }}_{k_{x}}^{2}=b_{x}\left[1+\frac{1}{Q}\left(\phi_{x,\mathrm{raw}}-1\right)\right].$$

Crow and Morton (1955) derived their equation for *Q* > 1, in which case Equations 7a and 7b provides the expected variance if offspring had been randomly subsampled until mean offspring number reached the target level ($b_{x}$). Waples (2002) showed that the same formula applies when *Q* < 1, in which case ${\hat{\sigma }}_{k_{x}}^{2}$ is the expected result if the same offspring distribution had been sampled more intensively.

This rescaling is done separately for each age or age‐at‐death, but using a common *Q* based on the total number of offspring sampled. For analysis of LRS, the above equation is valid after replacing *x* with *q* for age‐at‐death, and replacing the annual metrics with their respective lifetime analogues.

The following assumptions are made regarding the estimation process:

- Means and variances in offspring number are calculated with respect to the total number of adults alive at a given time. Including juveniles, which by definition cannot produce offspring, has predictable consequences related to zero‐inflation (Waples & Reed, 2023) but is not considered here.
- The researcher has known or estimated ages for all potential parents in the population, so the vector *N*_*x*_ is known or can be estimated.
- Empirical data provide sample estimates of relative fecundity at age (${\bar{k}}_{x}$) and age‐specific variance in offspring number ($s_{k_{x}}^{2}$), conditional on ${\bar{k}}_{x}$.
- From the vector of relative ${\bar{k}}_{x}$ estimates, the researcher can estimate the *b*_*x*_ values required to produce a stable population, using the expectation that Σ*l*_*x*_ *b*_*x*_ = 2. The vector *l*_*x*_ can be estimated from observed *N*_*x*_ values, or from independent data.
- The empirical estimate of *Q* to be used in rescaling the raw data is calculated as *Q* = Σ*l*_*x*_ ${\bar{k}}_{x}$/2.

### 2.6 Simulations

Computer simulations were run to confirm the accuracy of analytical results and to evaluate performance of the estimators. Main features of the simulations are summarized here; more details and computer code can be found in Supporting Information. All simulations were done in R (R Core Team, 2021) and modeled hypothetical populations with vital rates shown in Table 2. For both annual and lifetime reproduction, simulations were conducted for Case 1 and Case 2 sampling. In Case 1, the comprehensive sampling effort was fixed at *N*_Offspring_ = 2*N*_1_ (*Q* = 1), and *N*_1_ was varied across a 40‐fold range [50–2000] to evaluate effects of population size (and hence group size in the ANOVA analyses). For Case 2, *N*_1_ was set to either 100 or 500, and sampling effort was varied across an order‐of‐magnitude range (*Q* = [0.2,0.4,1,2]).

**TABLE 2:** Top: Vital rates for a hypothetical population having 5 age classes, constant survival (*v*_*x*_) at 50%/year, maturity at age 1, fecundity (*b*_*x*_) that is either constant, increases, or decreases with age, and variance in offspring number among individuals of the same age that is either random (*ϕ* = 1) or substantially overdispersed (*ϕ* = 10). *b*_*x*_ values have been scaled to values that will produce a stable population. Computer simulations used different combinations of these vital rates. Bottom: The range of population sizes modeled in the simulations. The vector of age‐specific *N*_*x*_ values (which are also the age‐class group sizes in the ANOVA analyses for annual reproduction) are determined by the relationship *N*_*x*_ = *l*_*x*_ *N*_*1*_, where *l*_*x*_ is cumulative survivorship through age *x*. In parentheses after the *N*_*x*_ values are the numbers of individuals (*D*_*q*_) that die after reaching age *q* but before reaching age *q* + 1. The *D*_*q*_ values are the group sizes in the ANOVA analyses of lifetime reproductive success. In this example, age at maturity is 1, so age (*x*) and age at death of adults (*q*) have the same range (1–5).

Age (*x*)	*v*_*x*_	Fecundity (*b*_*x*_)			*ϕ*	
		Constant	Increasing	Decreasing	Random	Overdispersed
1	0.5	1.03	0.56	1.24	1	10
2	0.5	1.03	1.12	0.99	1	10
3	0.5	1.03	1.69	0.74	1	10
4	0.5	1.03	2.25	0.50	1	10
5	0	1.03	2.81	0.25	1	10

*x* or *q*	*v*_*x*_	*l*_*x*_	*N*_*x*_ (*D*_*q*_)					
1	0.5	1	50 (25)	100 (50)	250 (125)	500 (250)	1000 (500)	2000 (1000)
2	0.5	0.5	25 (12)	50 (25)	125 (62)	250 (125)	500 (250)	1000 (500)
3	0.5	0.25	13 (7)	25 (12)	63 (32)	125 (62)	250 (125)	500 (250)
4	0.5	0.125	6 (3)	13 (7)	31 (15)	63 (32)	125 (62)	250 (125)
5	0	0.0625	3 (3)	6 (6)	16 (16)	31 (31)	63 (63)	125 (125)

## 3 RESULTS

### 3.1 Annual reproduction

#### 3.1.1 Parametric variance partitioning

To evaluate performance, it is necessary to establish what the true sums of squares are, which here are taken to be expected values in a stable population with demographics governed by parametric vital rates. These population parameters apply to a scenario in which all 2*N*_1_ offspring in a cohort have been sampled and assigned to parents, in which case *E* $({\bar{k}}_{x})=b_{x}$ and *E* $(\sigma_{k,x}^{2})=\phi_{x}b_{x}$ for all ages, and $E\left({\bar{k}}_{∙}\right)=\bar{b}$, where $\bar{b}=2N_{1}/N_{A}$ is the parametric mean offspring number across adults of all ages. The parametric sums of squares expectations are obtained by substituting these terms into Equations (1a), (1b), (1c) (see Appendix S1 for details):

(8a) $${\mathrm{SSE}}_{\text{parametric}}=\sum_{x=1}^{n}N_{x}\sigma_{k,x}^{2}=\sum_{x=1}^{n}N_{x}\phi_{x}b_{x}$$

(8b) $${\mathrm{SSB}}_{\text{parametric}}=\sum_{x=1}^{n}N_{x}{\left(b_{x}-\bar{b}\right)}^{2}.$$

The group‐size vector is *N*_*x*_ = *N*_*1*_ *l*_*x*_, so Equations 8a and 8b can be written as ${\mathrm{SSE}}_{\text{parametric}}=N_{1}\sum_{x=1}^{n}l_{x}\sigma_{k,x}^{2}$ and ${\mathrm{SSB}}_{\text{parametric}}=N_{1}\sum_{x=1}^{n}l_{x}{\left(b_{x}-\bar{b}\right)}^{2}$. Therefore,

$$\frac{{\mathrm{SSE}}_{\text{parametric}}}{{\mathrm{SST}}_{\text{parametric}}}=\frac{N_{1}\sum_{x=1}^{n}l_{x}\sigma_{k,x}^{2}}{N_{1}\left[\sum_{x=1}^{n}l_{x}\sigma_{k,x}^{2}+\sum_{x=1}^{n}l_{x}{\left(b_{x}-\bar{b}\right)}^{2}\right]}=\frac{\sum_{x=1}^{n}l_{x}\sigma_{k,x}^{2}}{\sum_{x=1}^{n}l_{x}\sigma_{k,x}^{2}+\sum_{x=1}^{n}l_{x}{\left(b_{x}-\bar{b}\right)}^{2}},$$

which means that the ratios ${\mathrm{SSE}}_{\text{parametric}}/{\mathrm{SST}}_{\text{parametric}}$ and ${\mathrm{SSB}}_{\text{parametric}}/{\mathrm{SST}}_{\text{parametric}}$ are independent of population size, provided that vital rates do not change with abundance.

#### 3.1.2 A null model

A variety of null models might be constructed for reproductive success (see Waples & Reed, 2023 for details), but a simple one in widespread use assumes that all potential parents function as a single Wright‐Fisher population, with random mating and equal expectations of reproductive success, *E*(*k*). Under those conditions, for all ages $b_{x}=E\left(k\right)=\bar{b}$ and $\phi_{x}=1$, which leads to

(9a) $${\mathrm{SSE}}_{\text{parametric},\text{null}}=\sum_{x=1}^{n}N_{x}E\left(k\right)=\bar{b}\sum_{x=1}^{n}N_{x}=\bar{b}N_{A}$$

(9b) $${\mathrm{SSB}}_{\text{parametric},\text{null}}=\sum_{x=1}^{n}N_{x}{\left[E\left(k\right)-E\left(k\right)\right]}^{2}=0.$$

As illustrated below, this null model can provide a useful reference point for analysis of empirical data.

#### 3.1.3 Estimation

For empirical data, Equations 8a and 8b are modified as follows (see Appendix S1 for details):

(10a) $$E\left({\mathrm{SSE}}_{\text{empirical}}\right)=E\left[\sum_{x=1}^{n}\sum_{i=1}^{N_{x}}{\left(k_{i,x}-{\bar{k}}_{x}\right)}^{2}\right]=\sum_{x=1}^{n}N_{x}E\left(s_{k_{x}}^{2}\right),$$

(10b) $$E\left({\mathrm{SSB}}_{\text{empirical}}\right)=\left(\frac{n-1}{n}\right)\sum_{x=1}^{n}E\left(s_{k_{x}}^{2}\right)\left(\text{random}\right)+\sum_{x=1}^{n}N_{x}{\left[E\left({\bar{k}}_{x}-{\bar{k}}_{∙}\right)\right]}^{2}\left(\text{deterministic}\right)$$

The first term in Equation 10b accounts for random contributions to ${\bar{k}}_{x}$, and (*n*–1)/*n* reflects the fact that the sample ${\bar{k}}_{x}$ values are constrained to have an overall weighted mean of ${\bar{k}}_{∙}$, so the number of degrees of freedom is one less than the number of groups (as it is for the between‐groups sum of squares in ANOVA).

Although group sizes are fixed constants, expectations for $s_{k_{x}}^{2}$ and $\left({\bar{k}}_{x}-{\bar{k}}_{∙}\right)$ depend on the sampling regime, as discussed below.

##### Case 1: Comprehensive sampling

With comprehensive sampling, variance rescaling is not necessary, so $E\left(s_{k_{x}}^{2}\right)=\phi_{x}b_{x}$ and $E\left({\bar{k}}_{x}-{\bar{k}}_{∙}\right)=E\left({\bar{k}}_{x})-E({\bar{k}}_{∙}\right)=\left(b_{x}-\bar{b}\right)$, and

(11a) $$E\left({\mathrm{SSE}}_{\text{comprehensive}}\right)=\sum_{x=1}^{n}N_{x}\phi_{x}b_{x}={\mathrm{SSE}}_{\text{parametric}}$$

(11b) $$\begin{array}{c} E\left({\mathrm{SSB}}_{\text{comprehensive}}\right)=\left(\frac{n-1}{n}\right)\sum_{x=1}^{n}E\left(s_{k_{x}}^{2}\right)+\sum_{x=1}^{n}N_{x}{\left(b_{x}-\bar{b}\right)}^{2}\\ =\left(\frac{n-1}{n}\right)\sum_{x=1}^{n}\phi_{x}b_{x}+{\mathrm{SSB}}_{\text{parametric}}. \end{array}$$

The unbiased estimator of the within‐age variance $\sigma_{k,x}^{2}$ is calculated from the raw data as

(12) $${\hat{\sigma }}_{k_{x}}^{2}=s_{k_{x}}^{2}=\frac{\sum_{i=1}^{N_{x}}{\left(k_{i,x}-{\bar{k}}_{x}\right)}^{2}}{N_{x}-1}.$$

An estimator of the overall with‐age sum of squares is then

(13) $${\hat{\mathrm{SSE}}}_{\text{comprehensive}}=\sum_{x=1}^{n}N_{x}{\hat{\sigma }}_{k_{x}}^{2}.$$

To estimate parametric SSB from empirical data it is necessary to subtract the expected value of the random component, leading to

(14) $$\begin{array}{c} {\hat{\mathrm{SSB}}}_{\text{comprehensive}}={\mathrm{SSB}}_{\mathrm{raw}}-E\left({\mathrm{SSB}}_{\text{random}}\right)\\ =\sum_{x=1}^{n}N_{x}{\left({\bar{k}}_{x}-{\bar{k}}_{∙}\right)}^{2}-{\left(\frac{n-1}{n}\right)\hat{\sigma }}_{\text{Within}}^{2} \end{array},$$

where ${\hat{\sigma }}_{\text{Within}}^{2}=\sum_{x=1}^{n}{\hat{\sigma }}_{k,x}^{2}$.

##### Case 2: Generalized sampling designs

For generalized sampling, expectations for the raw, empirical sums of squares as a function of *Q* and parametric vital rates are provided in Appendix S1. The next step is to develop unbiased estimators of the parametric variance components. Estimators used in this step are:

(15a) $${\hat{b}}_{x}=\left(\frac{1}{Q}\right){\bar{k}}_{1,x}$$

(15b) $$\hat{\bar{b}}=\left(\frac{1}{Q}\right){\bar{k}}_{∙}$$

and ${\hat{\sigma }}_{k_{x}}^{2}=s_{k_{x,\text{scaled}}}^{2}$ is computed as in Equation 12. These estimators are then used in Equation 13 to get the overall within‐group sum of squares estimator for generalized sampling:

(16a) $$\hat{\mathrm{SSE}}=\sum_{x=1}^{n}N_{x}{\hat{\sigma }}_{k_{x}}^{2}.$$

For SSB, it is simplest to rescale the vital rates before accounting for random differences in age‐specific fecundity (see Appendix S1 for details). The unbiased estimator of SSB is

(16b) $$\begin{array}{c} \hat{\mathrm{SSB}}={\mathrm{SSB}}_{\text{scaled}}-E\left({\mathrm{SSB}}_{\text{scaled},\text{random}}\right)\\ =\sum_{x=1}^{n}N_{x}{\left({\hat{b}}_{x}-\hat{\bar{b}}\right)}^{2}-\left(\frac{n-1}{n}\right)\sum_{x=1}^{n}{\hat{\sigma }}_{k,x}^{2}. \end{array}$$

#### 3.1.4 Simulations

**FIGURE 1:**
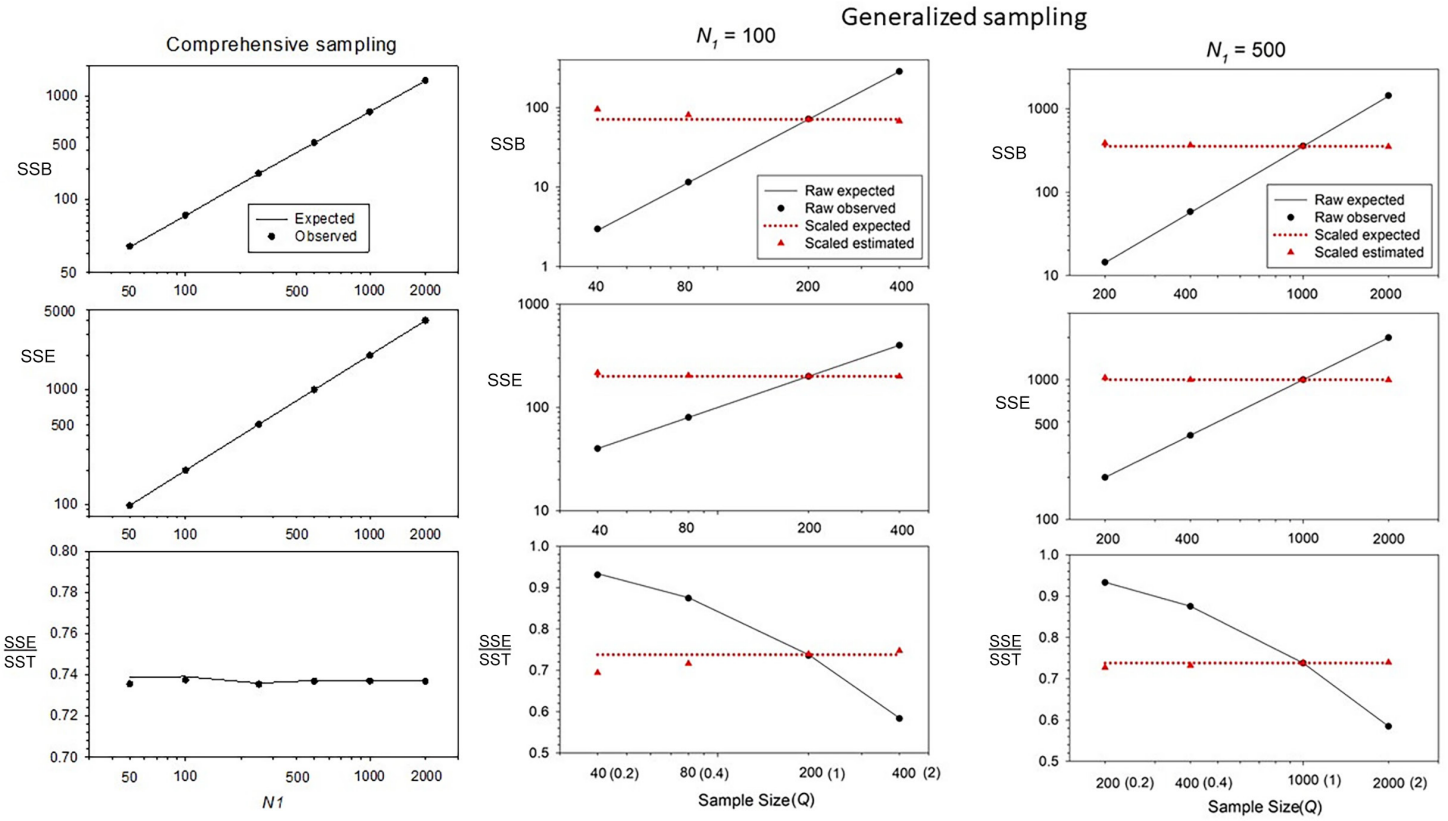
Results of simulations modeling annual reproduction in a hypothetical species for which fecundity increased linearly with age and variance in offspring number was random for individuals of the same age (all *ϕ*_*x*_ = 1; see Table 2). Left panels: Results for comprehensive sampling (Case 1) for a 40‐fold range of population sizes, as indexed by the *N*_1_ values on the *x* axis. See Figure S1 for comparable results for a null model with parametric expectations given by Equations 9a and 9b. Black circles and black solid lines show observed and expected results, respectively. Center and right panels: Results for generalized sampling (Case 2) for four different sampling intensities, indicated on the *x* axis by *Q* = *N*_Offspring_/(2*N*_1_) = [0.2, 0.4, 1, 2]. Black circles and black solid lines show observed and expected results, respectively, using the raw data; red triangles and red dotted lines show observed and expected results after rescaling the data per Equations 7a and 7b. Center panels show results for *N*_1_ = 100, and right panels show results for *N*_1_ = 500. In all cases, observed results are means across 10,000 replicates.

**FIGURE 2:**
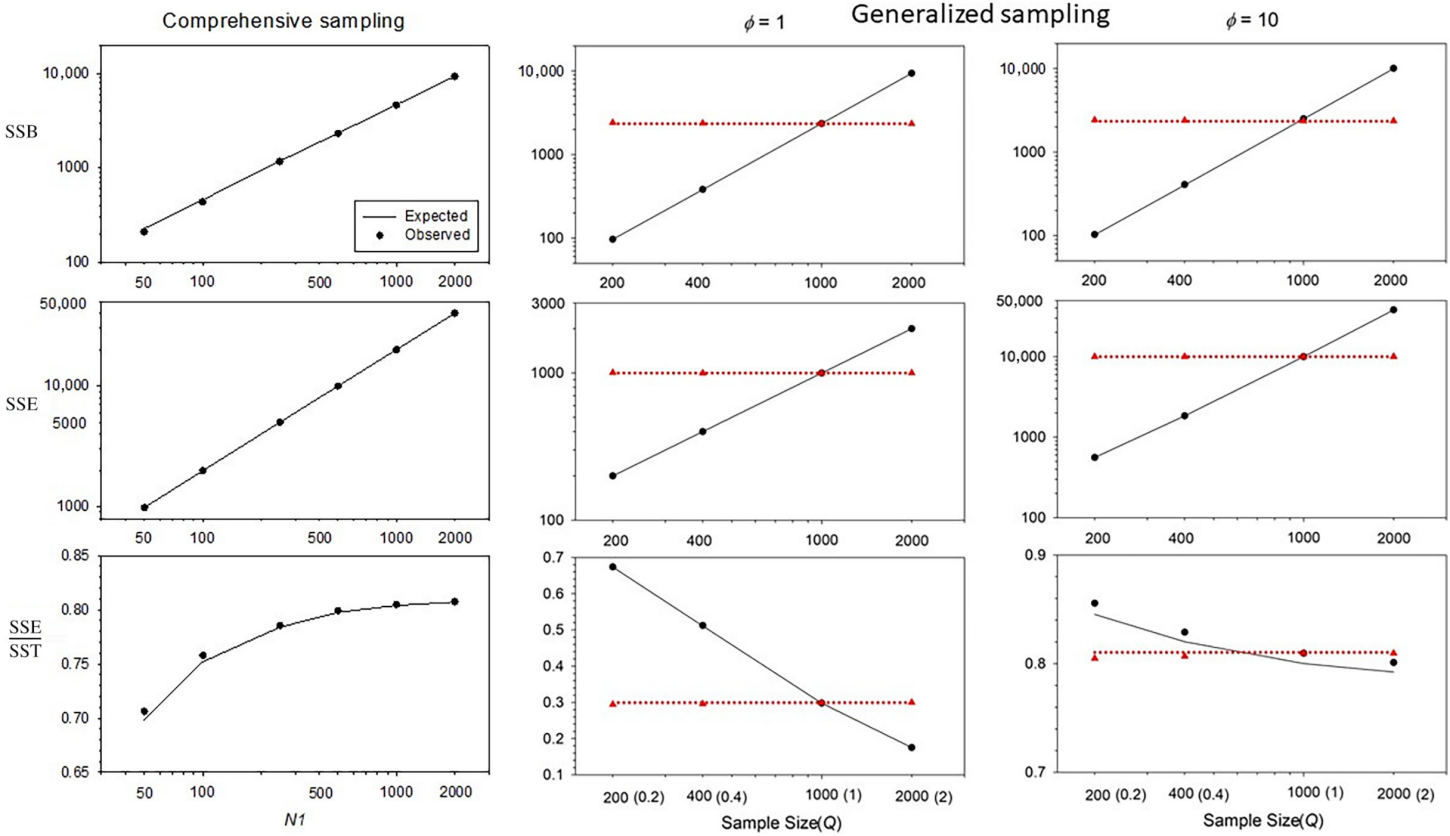
As in Figure 1, except showing results for lifetime reproductive success. For the Generalized sampling scenarios, center panels show results for *ϕ* = 1 and right panels show results for *ϕ* = 10, in both cases with *N*_1_ = 500 and increasing fecundity with age. For comprehensive sampling, *ϕ* = 1 with increasing fecundity with age.

Simulation results for annual reproduction are shown in Figure 1, Figures S1 and S2, and Box S1. Across the scenarios evaluated, the estimators of SSB, SSE, and SSE/SST were asymptotically unbiased for moderate to large sampling efforts and population sizes (hence group sizes). Biases that did occur for the smaller values of *Q* (where only 20–40% of the offspring were sampled) and group size (with *N*_1_ = 50, the oldest 2 age classes have only 3 and 6 individuals) generally applied to SSB but not SSE. Because the within‐age sum of squares is generally much larger than the between‐age sum of squares for annual reproduction, any bias to SSB causes proportionally less bias to the ratio SSE/SST, which is the primary quantity of interest. Details include the following:

- Parametric SSE and SSB both increase linearly with population/group size, but the random component to empirical SSB does not. As a consequence, any bias associated with adjusting for this random component becomes relatively less important as group size increases (see Box S1).
- Under a null model with no true differences in expected fecundity with age (all $b_{x}=\bar{b}$), $\hat{\mathrm{SSB}}$ was close to 0 for all scenarios, but with a slight tendency for underestimation (presumably because the correction for the random component was too large). This bias becomes smaller as sampling effort and group size increase (Figure S1).
- If fecundity increases with age (a common pattern in many species), $\hat{\mathrm{SSE}}$ remains unbiased but $\hat{\mathrm{SSB}}$ slightly overestimates parametric SSB, leading to a slight underestimate of SSE/SST. However, the resulting biases are small even for the smallest *Q* and *N*_1_ (the most extreme bias occurred with *Q* = 0.2 and *N*_1_ = 100, where estimated SSE/SST (0.78) was 4% higher than the parametric value, and this bias became negligible for *N*_1_ = 500; Figure 1). If fecundity decreases with age (as occurs with reproductive senescence), SSE is not affected but SSB is reduced compared to the increasing‐fecundity scenario, which increases SSE/SST (Figure S2).
- For generalized sampling, raw empirical SSE and SSB (represented by filled black circles in Figures 1 and 2) agreed closely with analytical expectations (solid black lines). Parametric expectations under comprehensive sampling are shown in dotted red lines, which intersect the trajectories of the raw data only for *Q* = 1, which represents comprehensive sampling. Red triangles show how close rescaled estimates come to these parametric expectations.
- Raw SSE and raw SSB both increase with sampling effort and hence mean offspring number, but they do so at different rates. As a consequence, the key ratio SSE/SST based on raw data can vary substantially based on sampling effort. For example, for the scenario in Figure 1 where fecundity increased with age, the proportion of the total sum of squares due to within‐age effects ranged from >0.9 for *Q* = 0.2 to <0.6 for *Q* = 2 (black symbols and solid black lines in the bottom panels for *N*_1_ = both 100 and 500). All of these samples were generated by a single (albeit hypothetical) population having one parametric set of vital rates, so this result illustrates the danger of using raw, unscaled data to draw inferences about variance partitioning.
- These results for the raw empirical data indicate that any biases associated with generalized sampling designs primarily arise during the variance‐rescaling process that uses the non‐linear Equations 7a and 7b. In this equation, 1/*Q* becomes a scaling factor that magnifies any small biases in the raw data.

#### 3.1.5 A worked example—Black bears from Michigan

During 2002–2010, Michigan state biologists estimated ages (from teeth) for over 2500 black bears (*Ursus americanus*) killed by hunters. This example focuses on data collected from genetic parentage analysis of a subset of bears, which yielded 221 matches of offspring to both parents (Moore et al., 2014) and allowed estimation of age‐specific vital rates (Table 3). Black bears can live at least 20 years, but individuals older than 10 are uncommon, so those were grouped into a single plus age class. Males mature at age 2; females generally mature age 3 and can have litters of up to 4–6 cubs. Primary sex ratio is even, but males have lower survival so adult females are more numerous.

**TABLE 3:** Variance partitioning analysis for seasonal reproduction by male black bears from Michigan.

	Raw data							Scaled data			Sums of squares			
Age	*v*_*x*_	*l*_*x*_	${\bar{k}}_{x}$	$s_{k_{x}}^{2}$	*l*_*x*_ ${\bar{k}}_{x}$	*ϕ*_*x*_	*N*_*x*_	${\hat{b}}_{x}$	${\hat{\sigma }}_{k_{x}}^{2}$	*ϕ*_*x*_	SSE	SSE_random_	SSB	$\Sigma k_{i}^{2}$
1	0.639	1.000	0.000	NA	0.0000	–	260	–	–	–	–	–	–	–
2	0.559	0.639	0.027	0.026	0.0173	0.963	166	0.541	0.1	0.3	23.1	89.8	65.6	72
3	0.670	0.357	0.030	0.032	0.0107	1.067	93	0.601	1.4	2.3	130.6	55.9	30.0	164
4	0.670	0.239	0.030	0.033	0.0072	1.100	62	0.601	1.8	3.0	112.0	37.3	20.0	134
5	0.670	0.160	0.062	0.069	0.0099	1.113	42	1.243	4.1	3.3	170.3	52.2	0.2	235
6	0.670	0.107	0.121	0.166	0.0130	1.372	28	2.425	20.5	8.5	574.1	67.9	44.1	739
7	0.670	0.072	0.156	0.321	0.0112	2.058	19	3.127	69.4	22.2	1318.9	59.4	72.8	1505
8	0.670	0.048	0.196	0.329	0.0095	1.679	13	3.929	57.4	14.6	745.7	51.1	98.9	946
9	0.670	0.032	0.238	0.354	0.0077	1.487	8	4.770	51.4	10.8	411.0	38.2	103.7	593
10	0.670	0.022	0.377	0.454	0.0082	1.204	6	7.556	38.5	5.1	230.9	45.3	244.7	574
11+	0.000	0.042	0.122	0.191	0.0052	1.566	11	2.445	30.2	12.3	331.8	26.9	17.9	398
Totals					0.0998	Ages 2 + 448		274.7		4048.5		524.0	698.1	5359

*Note*: Assuming constant production of *N*_1_ = 260 yearling males, these vital rates would produce an adult male population size of 448 age 2+ individuals. Columns under “Raw data” show estimates from field samples reported by Waples et al. (2018). Columns under “Scaled data” show variables that have been rescaled based on the estimated index of sampling intensity *Q* = 0.05. The “Sums of squares” columns show age‐specific within‐age (SSE) and between‐age (SSB) components based on scaled vital rates, and the $\Sigma k_{i}^{2}$ column (which shows age‐specific squared numbers of offspring per individual) is used to compute overall annual variance in offspring number and annual effective size, *N*_*b*_. See Table 1 for notation, Table S1 for comparable data for females, and text for explanation of the calculations.

Cumulative survival for males though age 10 was *l*_10_ = 0.022, and all older males were lumped into a single ‘plus’ (age 11+) age class, with overall Σ*l*_11+_ = 0.042. Assuming a fixed number of *N*_1_ = 260 yearling males each year, the rest of the age classes have *N*_*x*_ = *l*_x_*N_1_ individuals, with a total of *N*_*A*_ = 448 age 2+ adults. Sample estimates of age‐specific fecundity increased monotonically from ${\bar{k}}_{2}=0.027$ for age 2 to ${\bar{k}}_{10}=0.377$ for age 10, and (except for age 2) the associated sample variances ($s_{k_{x}}^{2}$) were all larger than the means, so raw *ϕ* values were > 1. Overall weighted mean sample offspring number is ${\bar{k}}_{∙}=\frac{\Sigma {\bar{k}}_{x}N_{x}}{N_{A}}=0.0584$. In a stable population with eqwual primar sex ratio (as applies to black bears) Σ*l*_x_*${\bar{k}}_{x}=2$, but for the raw data Σ*l*_x_*${\bar{k}}_{x}=0.0998$, so the index of sampling intensity was *Q* = 0.0998/2 = 0.05, indicating very sparse sampling.

To estimate parametric sums of squares, the first step is to rescale the raw data to values expected for a stable population. For each age, ${\hat{b}}_{x}$ and ${\hat{\sigma }}_{k,x}^{2}$ are computed from Equations 15a and 15b, and the overall mean is estimated as $\hat{\bar{b}}=\left(\frac{1}{Q}\right){\bar{k}}_{∙}=\frac{0.0584}{0.05}=1.17$. The rescaled age‐specific *ϕ*_*x*_ values are considerably higher (up to 22.2 for age 7 males), indicating that overdispersion is very substantial.

These rescaled variables are then used in Equations 16a and 16b to estimate parametric sums of squares (Table 3). Overall rescaled $\hat{\mathrm{SSE}}$ is 4048.5. Total SSB from the rescaled data is 698.1, of which $\left[\left(n-1\right)/n\right]*\Sigma {\hat{\sigma }}_{k_{x}}^{2}=0.9*274.7=247.2$ can be attributed to random differences in estimated age‐specific fecundity. Therefore, $\hat{\mathrm{SSB}}$ is 698.1–247.2 = 450.8. Of the total $\hat{\mathrm{SSE}}$, how much can be attributed to stochastic effects? Under a common null model, the distribution of offspring number within ages is Poisson, implying all *ϕ*_*x*_ = 1 and all ${\hat{\sigma }}_{k,x}^{2}={\hat{b}}_{x}$, and these terms sum to 524. Of the within‐ages component, therefore, only a small fraction (~13%) can be explained by random, Wright‐Fisher reproduction. The pie chart in Figure 4 depicts partitioning of the overall ANOVA sums of squares. Most (85%) of the total SST is due to within‐age effects, and most of that is attributable to greater‐than‐random variance in offspring number among males of the same age. Although fecundity increases sharply through at least age 10 in male black bears (Table 3), effects of this on variance in offspring number are dwarfed by within‐age effects.

The last column in Table 3 shows the parametric expectation for $\Sigma k_{i}^{2}$ for each age, calculated as in Equation 4. Across all ages, $\Sigma k_{i}^{2}$ is 5359, so the annual population variance is $\sigma_{k}^{2}=\frac{\Sigma k_{i}^{2}}{N_{A}}-{\left(\hat{\bar{b}}\right)}^{2}=\frac{5359}{448}-1.17^{2}=10.6$. Substituting values for the annual mean and variance in offspring number into Equation 3 produces *N*_*b*_ = 56.7^1^ and *N*_*b*_/*N*_*A*_ = 56.7/448 = 0.126—so in this example annual effective size of male black bears is one‐eighth of the number of adult males. Under a null Wright‐Fisher model, $E\left(\sigma_{k}^{2}\right)=\hat{\bar{b}}$, leading to *N*_*b*_/*N*_*A*_ = 1, so all the reductions in the *N*_*b*_/*N*_*A*_ ratio can be attributed to greater‐than‐random variance within and between ages. From above, the greater‐than‐random component to SST is SSE_>random_ + SSB_>random_ = 3524 + 451 = 3975, of which 88.7% is from within‐age effects and 11.3% from between‐age effects. The total reduction in the *N*_*b*_/*N*_*A*_ ratio is 87.4%, of which the fraction 0.887, or 77.4%, is attributable to overdispersed within‐age variance, and the remaining 9.9% reduction is due to systematic changes in fecundity with age.

For the annual male black bear data, Crow's *I* is $\frac{\sigma_{k}^{2}}{{\left(\hat{\bar{b}}\right)}^{2}}=\frac{10.6}{1.17^{2}}=7.74.$ Of this, 1/$\hat{\bar{b}}$ = 0.85 can be attributed to random reproductive success, so the greater‐than‐random component of the Opportunity for Selection is $\Delta_{I}=I-\frac{1}{\hat{\bar{b}}}=7.74-0.85=6.89.$ These non‐random contributions to the Opportunity for Selection can be partitioned in the same way as the reductions in *N*_*b*_/*N*_*A*_: 88.7% of $\Delta_{I}$ = 6.11 is attributable to overdispersded within‐age variance, and the remainder (0.78) to differences in fecundity with age.

Table S1 replicates these analyses for female black bears, for which both changes in fecundity with age and age‐specific *ϕ*_*x*_ are smaller than in males. The smaller magnitudes of nonrandom SSE and SSB cause a smaller reduction in effective size in females, so the *N*_*b*_/*N*_*A*_ ratio (0.271) is a bit over twice as large as for males. However, females mimic males in that the overwhelming majority of reductions in *N*_*b*_/*N*_*A*_ are due to overdispersed within‐age variance rather than changes in fecundity with age. Similarly, in both sexes the non‐random component of the Opportunity for Selection is dominated by within‐age effects. The patterns are displayed visually in Figure 4.

### 3.2 Lifetime reproductive success

The ANOVA sums of squares for lifetime SSB•, SSE•, and SST• in Equations (2a), (2b), (2c) are superficially similar in form to Equations (1a), (1b), (1c) for annual reproduction, but with an important difference: for analysis of LRS, groups are defined by age‐at‐death, which means that all groups with *q* > 1 record cumulative LRS over two or more years. As shown in Supporting Information, a consequence of this is that the group‐specific terms for SSE• take the form

$$\mathrm{SSE}{•}_{q}=D_{q}\left[\mathrm{var}\left(k_{i,1}\right)+\mathrm{var}\left(k_{i,2}\right)+\ldots \mathrm{var}\left(k_{i,q}\right)+2\sum_{j<k}^{q}\mathrm{cov}\left(k_{i,j},k_{i,k}\right)\right].$$

This means that within‐group variances are simple additive functions of age‐specific variances only if an individual's reproduction at one age does not affect its survival or reproduction at any subsequent age. That in fact is a common assumption in modeling age‐structured populations (e.g., Felsenstein, 1971; Waples et al., 2011), and to make the analytical expectations tractable that assumption is adopted here.

In many species, however, these covariance terms are not expected to be 0. Persistent individual differences occur when certain individuals are consistently above or below average for their age and sex at producing offspring (Lee et al., 2011), and these persistent differences lead to positive covariances in offspring production over time. Conversely, negative covariances occur when reproduction by an individual in one time period negatively affects its reproduction in subsequent time periods. Transient negative effects of this type are found in many species that exhibit skip or intermittent breeding (Shaw & Levin, 2013; Waples & Antao, 2014), and permanent negative effects can occur if reproduction adversely affects survival (e.g., McCleery et al., 1996). These temporal covariances do not affect calculation of SSE• from empirical data using Equation 2a, but to the extent that they do occur they will be reflected in the magnitude of the within‐group sum of squares and will affect agreement with expectations based on the simpler model.

SSB• deals with group means rather than individuals and is not sensitive to the temporal correlations of individual reproductive success that affect SSE•. However, the group means ${\bar{k•}}_{q.}$ are cumulative sums of LRS over time and hence are positively correlated. For example, ${\bar{k•}}_{2}={\bar{k}}_{1}+{\bar{k}}_{2}$ and ${\bar{k•}}_{3}={\bar{k}}_{1}+{\bar{k}}_{2}+{\bar{k}}_{3}$ share terms for mean LRS for individuals that die at ages 2 and 3. Furthermore, the weighted sums of squares and the weighted variance are both affected by the correlation between the patterns of change in group sample size and fecundity change with age (see Box S2 for details).

#### 3.2.1 Parametric variance components

As with annual reproduction, parametric values are considered to be expected values in a stable population in which all lifetime offspring have been assigned to the *N*_1_ potential parents in a cohort. Since the population is stable, overall mean offspring number for the cohort is ${\bar{k•}}_{∙}=2$.

Under those conditions, the parametric sums of squares for LRS are (see Appendix S1 for details):

(17a) $$\mathrm{SSE}{•}_{\text{parametric}}=\sum_{q=1}^{n}D_{q}\sum_{x=1}^{q}\phi_{x}b_{x},$$

(17b) $$\mathrm{SSB}{•}_{\text{parametric}}=\sum_{q=1}^{n}D_{q}*{\left(\left(\sum_{x=1}^{q}b_{x}\right)-2\right)}^{2}.$$

Two factors contribute to the squared‐difference terms in Equation 17b: (1) changes in fecundity with age, which modulate the magnitude of $\sum_{x=1}^{q}b_{x}$, and (2) differences among individuals in age‐at‐death (longevity, indexed by *q*). These two factors can be separated by holding fecundity constant with age, which eliminates factor 1, so the residual SSB• can all be attributed to variation in longevity. If all $b_{x}=\bar{b}$, then expected LRS for an individual that dies at age *q* is $q\bar{b}$, so the above equation simplifies to

(17c) $$\mathrm{SSB}{•}_{\text{parametric},\text{longevity}}=\sum_{q=1}^{n}D_{q}{\left(q\bar{b}-2\right)}^{2},$$

while the remainder represents the between‐age component of parametric SSB•.

#### 3.2.2 Estimation

##### Case 1: Comprehensive sampling

Following the framework used for annual reproduction, a logical estimator of the overall with‐group sum of squares for lifetime reproduction is

(18a) $$\hat{\mathrm{SSE}•}=\sum_{q=1}^{n}D_{q}{\hat{\sigma }}_{k•,q}^{2},$$

where ${\hat{\sigma }}_{k•,q}^{2}$ is the unbiased estimate of the variance within each group.

For comprehensive empirical data,

$$E\left(\mathrm{SSB}{•}_{\text{comprehensive}}\right)=\frac{n-1}{n}\sum_{q=1}^{n}{\hat{\sigma }}_{k•,q}^{2}+\sum_{q=1}^{n}D_{q}*{\left(\left(\sum_{x=1}^{q}b_{x}\right)-2\right)}^{2},$$

and an unbiased estimator for SSB• is

(18b) $${\hat{\mathrm{SSB}•}}_{\text{comprehensive}}=\mathrm{SSB}{•}_{\mathrm{raw}}-\frac{n-1}{n}\sum_{q=1}^{n}{\hat{\sigma }}_{k•,q}^{2}.$$

The estimator $\hat{\mathrm{SSB}•}$ accounts for the same two factors that contribute to parametric SSB•: changes in fecundity with age, and differences in longevity. ${\hat{\mathrm{SSB}•}}_{\text{longevity}}$ can be calculated from Equation 17c using the estimator of overall mean annual offspring number ($\hat{\bar{b}}$) from comprehensive sampling.

##### Case 2: Generalized sampling designs

As before, we consider sampling at level *Q* compared to comprehensive sampling and first develop an expectation for the raw sums of squares as a function of *Q* (see Appendix S1 for those results). Next we want to rescale the raw (empirical) variances to expected values under comprehensive sampling. With analogy to Equations 7a, 7b, 15a and 15b,

(19a) $$\hat{{\Sigma b}_{x}}=\left(\frac{1}{Q}\right){\bar{k•}}_{q}$$

(19b) $$\hat{\bar{b•}}=\left(\frac{1}{Q}\right){\bar{k•}}_{∙}$$

(19c) $${\hat{\sigma }}_{•q}^{2}=\hat{{\Sigma b}_{x}}\left[1+\frac{1}{Q}\left(\frac{s_{k•,q}^{2}}{{\bar{k•}}_{q}}-1\right)\right],$$

Leading to

(20a) $$\hat{\mathrm{SSE}•}=\sum_{q=1}^{n}D_{q}{\hat{\sigma }}_{•q}^{2}.$$

As with annual reproduction, to estimate parametric SSB• under generalized sampling, the first step is to rescale the age‐specific vital rates, producing:

$$E\left(\mathrm{SSB}{•}_{\text{scaled}}\right)=\frac{n-1}{n}\sum_{q=1}^{n}D_{q}*{\hat{\sigma }}_{k•,q}^{2}+\sum_{q=1}^{n}D_{q}{\left(\left(\sum_{x=1}^{q}\hat{\bar{b}}\right)-2\right)}^{2},$$

and

(20b) $$\hat{\mathrm{SSB}•}=\mathrm{SSB}{•}_{\text{scaled}}-\frac{n-1}{n}\sum_{q=1}^{n}D_{q}*{\hat{\sigma }}_{k•,q}^{2}.$$

#### 3.2.3 Simulations

In many respects, simulation results for lifetime reproduction paralleled those for annual reproduction:

- $\hat{\mathrm{SSE}•}$ is essentially unbiased even for small group sizes and low sampling effort;
- $\hat{\mathrm{SSB}•}$ shows some minor bias for low *N*_1_ that largely disappears with larger group sizes;
- Except under comprehensive sampling, raw LRS data require rescaling to produce unbiased estimates of parametric sums of squares.

A few important differences are also worth noting. First, under the null model with constant fecundity, SSB• does not have an expectation of 0, as it does for annual reproduction. Individuals that die at different ages differ in number of opportunities to participate in reproduction and hence have different expectations of LRS. Results for SSB• for the null model thus can all be attributed to variation in longevity (Figures S2 and S4). All else being equal, therefore, SSB• makes a relatively larger contribution to overall SST• than annual SSB does to SST.

A second and related point is that the pattern of change (if any) in fecundity with age has a strong effect on SSB•. This does not lead to bias, because these effects are fully accounted for in Equations 18b and 20b. However, as shown in Figure S4 and Box S2, if fecundity declines with age (as it can with reproductive senescence), total SSB• can be less than would be expected if fecundity were constant (i.e., total SSB• < $\mathrm{SSB}{•}_{\text{longevity}}$). The interpretation in this case would be that the pattern of between‐age differences in fecundity reduces overall SSB• compared to what it would be if SSB• only reflected differences in longevity.

**FIGURE 3:**
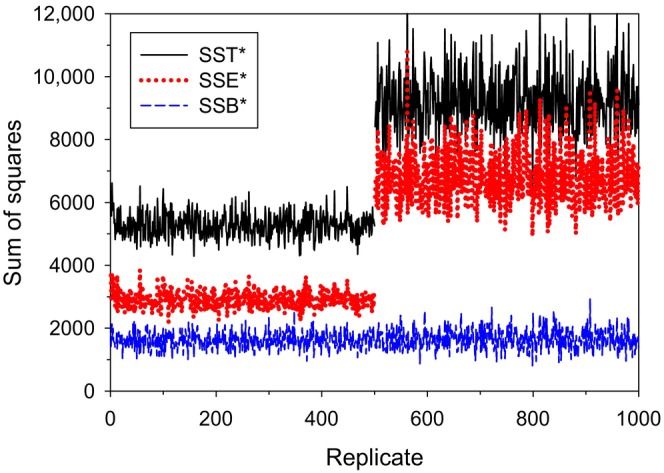
Results of simulations of LRS using the vital rates shown in Table 2, with fecundity that increased with age and moderately overdispersed variance in reproductive success (all *ϕ*_*x*_ = 3). The *y* axis shows the sum of squares for the three variance components, with the groups defined by age‐at‐death. On the *x* axis, in replicates 1–500 parental weights were shuffled each year, so there were no persistent individual differences in reproductive success. In replicates 501–1000, the same parental weights were retained across individual lifetimes, which introduced positive correlations in realized reproductive success across time; this had no effect on SSB• but sharply increased SSE• and hence SST•.

Finally, positive or negative correlations in reproduction over time can have a strong influence on SSE•, whereas they have no effect on annual SSE because the latter considers only one time period. The example in Figure 3 simulated a population using a generalized Wright‐Fisher model (Waples, 2022), where individuals were allowed to have unequal probabilities of producing offspring, as indicated by a vector of parental weights, **W** (see Appendix S1 for details). Randomly scrambling the weights each year satisfies the assumption of independence across time, producing results shown in the first half of the replicates in Figure 3. Allowing individuals to retain their weights throughout their lifetimes (second half of the replicates) creates persistent individual differences and positive correlations in individual reproductive success over time, which substantially increase SSE• (and hence SST•) but have no effect on SSB•.

#### 3.2.4 Worked example – great tits

The great tit (*Parus major*) is a woodland passerine with a wide distribution in Europe, including the UK. Four Dutch populations have been intensively monitored since the 1950s (Visser et al., 2021). Study sites are wooded areas fitted with an abundance of nest boxes; each year, every female that lays a clutch is captured and her ID recorded. Chicks are banded before fledging to allow tracking in the future. Data used here pertain to the cohort of birds at the Hoge‐Veluwe site that matured at age 1 in 1980.

Although great tits occasionally live to 8–9 years, life expectancy is 2 years or less. In this cohort, females reproduced only at ages 1–4, so for analysis of LRS we consider *n* = 4 groups with ages‐at‐death *q* = 1–4. Raw data are first tabulated into a matrix with one row per female:

**Table:** 

ID	Age1	Age2	Age3	Age4	LRS	*q*
1	0	0	NA	NA	0	2
2	1	1	0	NA	2	3
3	0	NA	NA	NA	0	1
5	1	NA	NA	NA	1	1
6	0	NA	2	3	5	4

Columns 2–5 show the number of age‐1 recruits produced by each female at each age, with LRS being the total. A “0” indicates the bird was recorded attempting a nest that year but produced no recruits that were recorded in subsequent years; “NA” indicates the bird was not observed that year. Age‐at‐death (*q*) was taken to be the oldest age with reproductive success data. In this matrix, female 1 died after age 2 without producing any recruits, female 2 produced 1 recruit at ages 1 and 2 before producing a clutch (but no surviving recruits) at age 3, and female 6 produced a clutch but no recruits at age 1, was not observed at age 2, and then produced 2 and 3 recruits, respectively, at ages 3 and 4, so its LRS is 5.

Females can be grouped by age‐at‐death to produce results shown in Table 4. The cohort includes Σ*D*_*q*_ = 81 females, with *D*_1_ = 48 dying after reproducing at age 1, and just 5 that reproduced at age 4. Reconstructed adult census size, based only on data for this cohort, is Σ*N*_*x*_ = 138 females. Columns ${\bar{k•}}_{q}$ and $s_{k•,q}^{2}$ are mean and unbiased sample variance in LRS for each age‐at‐death. The sample variance is slightly overdispersed for *q* = 1–3 (${\mathrm{raw\phi}}_{q}=\frac{s_{k•,q}^{2}}{{\bar{k•}}_{q}}>1$), but $s_{k•,4}^{2}\ll {\bar{k•}}_{4}$, indicating substantial underdispersion in the oldest age‐at‐death group. This group includes only 5 individuals that, by luck or pluck, all left 3–5 total offspring. The 81 members of the cohort produced a total of 61 lifetime offspring, so overall mean sample LRS for the cohort is ${\bar{k•}}_{∙}=$ 61/81 = 0.753, much less than mean LRS expected for a stable population (2), so *Q* = 0.753/2 = 0.376. Although birds that build nests within the study area are exhaustively sampled, reproduction also occurs in surrounding woods, so subsequent sampling of dispersing recruits born within the study area is not comprehensive.

**TABLE 4:** ANOVA analysis of lifetime reproductive success in the cohort of female great tits that matured at age 1 in 1980 in the Hoge‐Veluwe site in the Netherlands.

*x,q*	*D*_*q*_	*N*_*x*_	Raw LRS data			Scaled data			Sums of squares			
			${\bar{k•}}_{q}$	$s_{k•,q}^{2}$	*ϕ*_*q*_	$\Sigma \hat{b_{x}}$	${\hat{\sigma }}_{•q}^{2}$	*ϕ*_*q*_	SSE•	SSE•_random_	SSB•	SSB•_long_
1	48	81	0.46	0.51	1.11	1.22	1.58	1.30	75.7	56.3	29.5	32.8
2	14	33	0.50	0.58	1.15	1.33	1.87	1.41	26.2	32.9	6.3	1.7
3	14	19	0.93	1.46	1.57	2.47	6.21	2.52	87.0	49.3	3.1	32.4
4	5	5	3.80	1.20	0.32	10.09	0.00	0.00	0.0	23.5	327.5	36.3
	81	138					9.7		188.9	162.0	366.4	103.2

*Note*: The “*D*_*q*_” column shows the distribution of ages‐at‐death for the 81 members of the cohort. Columns under “Raw LRS data” show estimates of LRS metrics based on field samples of age‐1 recruits. Columns under “Scaled data” show variables that have been rescaled based on the estimated index of sampling intensity *Q* = 0.376. For *q* = 4, rescaling the raw $s_{k•,q}^{2}$ produced a negative result, so ${\hat{\sigma }}_{•4}^{2}$ was recorded as 0. The “Sums of squares” columns show within‐age‐at‐death (SSE•) and between‐age‐at‐death (SSB•) components. See Table 1 for notation, and text for explanation of the calculations.

Rescaling the raw data produces estimates of parametric vital rates, based on the relationships that ${\hat{b}}_{q}=\frac{{\bar{k•}}_{q}}{Q}$ and ${\hat{\sigma }}_{k•,q}^{2}={\hat{b}}_{q}\left[1+\frac{1}{Q}\left(\frac{s_{k•,q}^{2}}{{\bar{k•}}_{q}}-1\right)\right]$. Finally, estimates of parametric sums of squares are made as follows: $\hat{\mathrm{SSE}•}=\sum_{q=1}^{n}{\hat{\mathrm{SSE}•}}_{q}=\sum_{q=1}^{n}D_{q}{\hat{\sigma }}_{k•,q}^{2}=188.9$. If all *ϕ*_*x*_ were 1, *E*(SSE•_random_) = 162, so most of the empirical SSE• can be explained by random reproduction. For the between‐group sum of squares, SSB• is 366.4, from which we subtract $\left[\frac{n-1}{n}\right]*\sum_{q=1}^{n}\sigma_{k•,q}^{2}=0.75*9.7=7.2$ to account for stochasticity, leaving $\hat{\mathrm{SSB}•}=359.1$. To evaluate how much of this can be explained by random variation in longevity, replace all *b*_*x*_ by $\hat{\bar{b}}=2N_{1}\Sigma qD_{q}=1.17$. The result (${\hat{\mathrm{SSB}•}}_{\text{longevity}}=103.2$) is about 30% of total $\hat{\mathrm{SSB}•}$, with the remainder attributed to differences in fecundity with age. The estimate of the total parametric sum of squares is $\hat{\mathrm{SST}•}=\hat{\mathrm{SSE}•}+\hat{\mathrm{SSB}•}=548.0$, of which a bit over a third (34.5%) is due to within‐group effects. Most of the latter is attributable to random variation in reproductive success among same‐age individuals (hence, overdispersion within ages is modest). Effects of persistent individual differences in reproductive success would appear in SSE•. To evaluate this, I computed an index (*ρ*_*α,α+*_) proposed by Waples (2023), which is the correlation across individuals between the number of offspring produced at the age at maturity (*α*) and total offspring produced during the rest of their lifetimes. Persistent individual differences in reproductive success are expected to produce positive values of *ρ*_*α,α+*_, whereas negative values can be caused by skip breeding or tradeoffs between reproduction and survival. The correlation was not significant (*ρ*_*α,α+*_ = −0.137, *p* = .22, *n* = 81 for a two‐tailed test), suggesting that effects, if any, were relatively minor.

To evaluate uncertainty in the variance partitioning, annual data for the 81 females in the cohort were bootstrapped 10,000 times and the parametric sums of squares were re‐estimated from each replicate (Figure 5). The 95% bootstrapped CI for $\hat{\mathrm{SSE}•}$ extends well below the null expectation, so there is no overall evidence for within‐age overdispersion compared to random Poisson variance. The bootstrap CI for $\hat{\mathrm{SSB}•}$ is wide but the lower bound (124) is larger than the null expectation that all between‐age‐at‐death differences in LRS can be attributed entirely to variation in longevity—which is consistent with reports of some modest changes in fecundity with age in this species (Bouwhuis et al., 2012). The median bootstrap ratio $\hat{\mathrm{SSB}•}/\hat{\mathrm{SST}•}=0.68$ agreed well with the conclusion from the original data that about two thirds of the total lifetime variance was due to SSB•, but the empirical 95% CI was wide (0.34–0.87), so the exact partitioning is uncertain. Much of this uncertainty can be attributed to the small number (5) of birds that lived to age 4, all of which had high LRS; how many of those birds were selected in each bootstrap replicate therefore had a large effect on results.

With overlapping generations, *N*_*e*_ is proportional to generation length (Equation 5), so parsing factors contributing to the *N*_*e*_/*N* ratio is more complicated than it is for annual *N*_*b*_/*N*. If all *N*_1_ members of the cohort had random variation in LRS, then *E*(${\sigma_{k}^{2}}_{•}$) = mean (LRS) = 2, in which case Equation 5 would simplify to $N_{e}=N_{1}T$, which is the number of newborns entering the population in a generation (Hill, 1972). Accordingly, we focus on factors that elevate ${\sigma_{k}^{2}}_{•}$ compared to the null expectation. The constant‐*N* scaled estimate of the variance of LRS is ${{\hat{\sigma }}_{k}^{2}}_{•}=6.13$, about 3 times as large as the null expectation. The 3 relevant factors are overdispersed variance in reproductive success among same‐age individuals, systematic changes in fecundity with age, and variation in longevity. Of these, changes in fecundity with age are by far the most important (~3 times the longevity effect), and within‐age effects are only of minor importance.

The Opportunity for Selection metric for LRS, adjusted to account for random contributions, is designed to be calculated from raw data (Waples & Reed, 2023). The sample mean LRS is 0.753 and the sample variance is 1.34, so the raw OFS is $I•=\frac{{s_{k}^{2}}_{•}}{{{\bar{k•}}_{∙}}^{2}}=\frac{1.34}{0.567}=2.36$. From this we subtract expected contributions from sampling offspring (which is the inverse of mean LRS) and a term to account for random variation in longevity, which is independent of sampling effort. This latter term (from Equations 10a and 10b in Waples & Reed, 2023) is:

$$E\left(I{•}_{\text{longevity}}\right)=\frac{\sum_{q=1}^{n}D_{q}*{\left(q-\bar{q}\right)}^{2}}{\;{\bar{q}}^{2}\sum_{q=1}^{n}D_{q}},$$

where $\bar{q}=\sum_{q=1}^{n}{\mathrm{qD}}_{q}/\sum_{q=1}^{n}D_{q}$ is the mean age at death. For the great tit data, $\bar{q}=1.70$ and $E\left(I{•}_{\text{longevity}}\right)=0.32$. The net OFS metric that represents greater‐than‐random variance in LRS is

$$\Delta_{I•}=2.36-\frac{1}{0.753}-0.32=0.713.$$

As with reductions to effective size, almost all of this $\Delta_{I•}$ can be attributed to between‐age effects.

## 4 DISCUSSION

Important points that emerge from results presented above can be summarized as follows:

- The ANOVA sums of squares formulas in Equations (1a), (1b), (1c) and Equations (2a), (2b), (2c) do not require any assumptions about demographic or population dynamic processes and can be used with any empirical datasets that include numbers of offspring produced by each potential parent in each time period.
- Robust estimates of parametric within‐ and between‐group sums of squares also provide robust estimates of the proportions of the total variance in offspring number arising from these two sources of variation.
- For a given age structure (relative age‐group sizes determined by the cumulative survivorship vector *l*_*x*_), variance partitioning is independent of *N*, which means that randomly subsampling potential parents produces unbiased estimates of the variance partitioning (Figure S5). However, because the mean and variance of offspring number are positively correlated, variance partitioning is NOT independent of the fraction of offspring sampled. Under generalized sampling, empirical means and variances can be rescaled using an index of sampling intensity (*Q*) to allow meaningful comparisons across studies. *Q* can be estimated directly from the raw data.
- Estimators of parametric sums of squares developed here are asymptotically unbiased, with modest biases to $\hat{\mathrm{SSB}}$ and $\hat{\mathrm{SSB}•}$ when some group sizes are <10 and/or sampling is very sparse.

**FIGURE 4:**
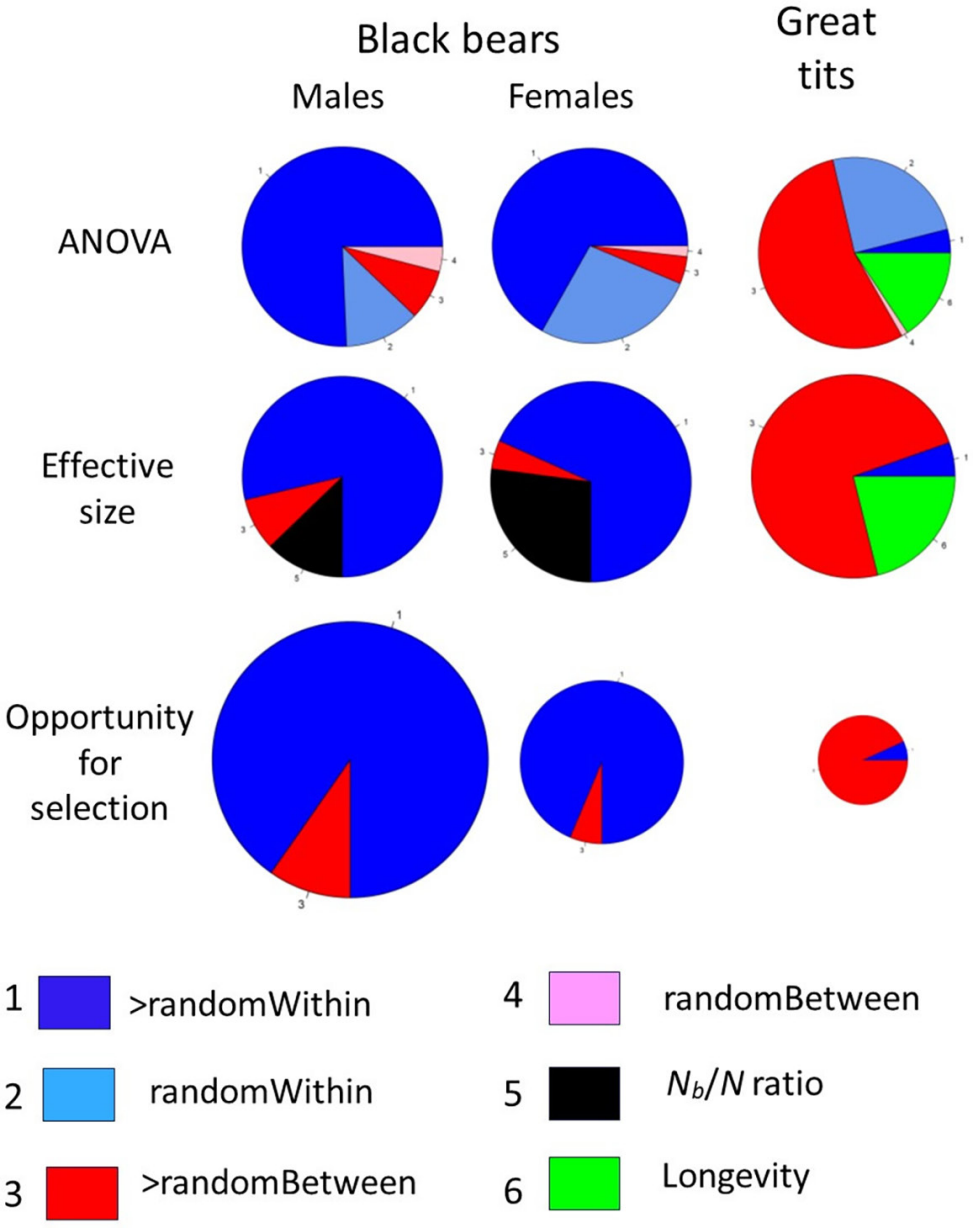
Graphical depiction of the partitioning of components of variation in offspring number for annual reproduction in black bears (based on data in Table 3 and Table S1) and lifetime reproduction in great tits (based on data in Table 4). In the ANOVA panels, the area of each pie is overall SST for rescaled data, and colored segments depict the relative contributions of random and greater‐than‐random within‐age (SSE) and between‐age (SSB) components; for LRS in great tits, the relative contribution from random variation in longevity is also shown. The area of each annual effective size pie for black bears represents unity; the black sector represents the *N*_*b*_/*N* ratio, and the blue and red sectors show reductions in *N*_*b*_/*N* that are attributable to within‐age and between‐age effects, respectively. The lifetime effective size pie for great tits shows the relative contributions of within‐age, between‐age, and longevity effects on variance in LRS. The Opportunity for Selection panels show the magnitude of OFS that exceeds that expected under a null model of random variation in reproductive success. The area of each pie is proportional to $\Delta_{I}$ (or $\Delta_{I•}$ for LRS in the great tit), and the relative sizes of the within‐age and between‐age effects are shown by the colored segments. Numbers next to colors in the legend correspond to numbered segments in the pie charts.

Without in any way suggesting that the topic considered here is as consequential as the one Lewontin tackled in his landmark 1972 paper, some important parallels can be identified between his apportionment of human genetic diversity and partitioning of variance in offspring number. A major point of Lewontin's paper was that the genetic differences most people were focusing on (between races, or geographic populations within races) are dwarfed by the ‘other’ ~85% of molecular genetic variation that is found among individuals within those groups. The situation is similar for partitioning variance in offspring number, where the within‐age sum of squares for annual reproduction (SSE) generally dominates the overall variance, even when fecundity changes sharply with age and variance within ages is Poisson (Figure S1). Published literature, however, consistently focuses primarily on the between‐age component (indexed by *b*_*x*_ values from a life table) and largely ignores the within‐age component. That is akin to ignoring all but the small red sectors in the black bear pie charts in Figure 4—that is, the ‘other’ 85+% of the total variation. Notably, SSE is also ≫SSB (and to a proportionally greater degree) for female black bears, which is a bit surprising, given that in most species males are expected to have higher reproductive variance. SSE is lower in female black bears than in males, but SSB is as well (and to a proportionally greater degree), leading to the net result that the within‐age component is relatively more important in females.

The typical outcome of variance partitioning is somewhat different for lifetime reproduction. Mortality inevitably creates disparities in individual longevity, which increase SSB• and tend to make the variance partitioning more even. Still, the within‐age component (SSE•) generally is fairly substantial and can dominate if within‐age variance is overdispersed (*ϕ* > 1; Figure 2).

The two worked examples illustrate some of the vagaries of dealing with empirical data for natural populations. Although the black bear data were collected during an intensive study that lasted most of a decade, this represents less than half of the maximum lifespan for the species, so analysis of LRS was not feasible. The 221 parent‐offspring matches also represented a small fraction of the estimated numbers of potential parents that might have produced matches, so effective sampling effort was very sparse (estimated at *Q* = 5% for males). Nevertheless, this sparse sampling was sufficient to demonstrate that, in both sexes, within‐age effects account for most of the overall variance in annual offspring number. Because the experimental design required combining estimates for reproduction in different years, the estimated SSE component includes a year effect of unknown magnitude. With more extensive data, one could estimate and account for this year effect (as done for example by Engen et al., 2005, 2010, who treated it as a random environmental effect).

Somewhat ironically, although breeding pairs of great tits are exhaustively sampled each year within the study area (as are eggs and fledglings they produce), the variance partitioning had a relatively high degree of uncertainty. Two factors are primarily responsible for this result. First, surviving birds can return to breed in the surrounding woods, so sampling of offspring at the recruit (age 1) stage is not exhaustive (estimated here at *Q* = 37% for the cohort in question). Second, the population is relatively small, in the sampled cohort only 5 females lived to reproduce at age 4, and these birds all had high and nearly identical LRS. These five individuals were highly influential to the variance partitioning, as reflected in the wide range of bootstrapped results.

**FIGURE 5:**
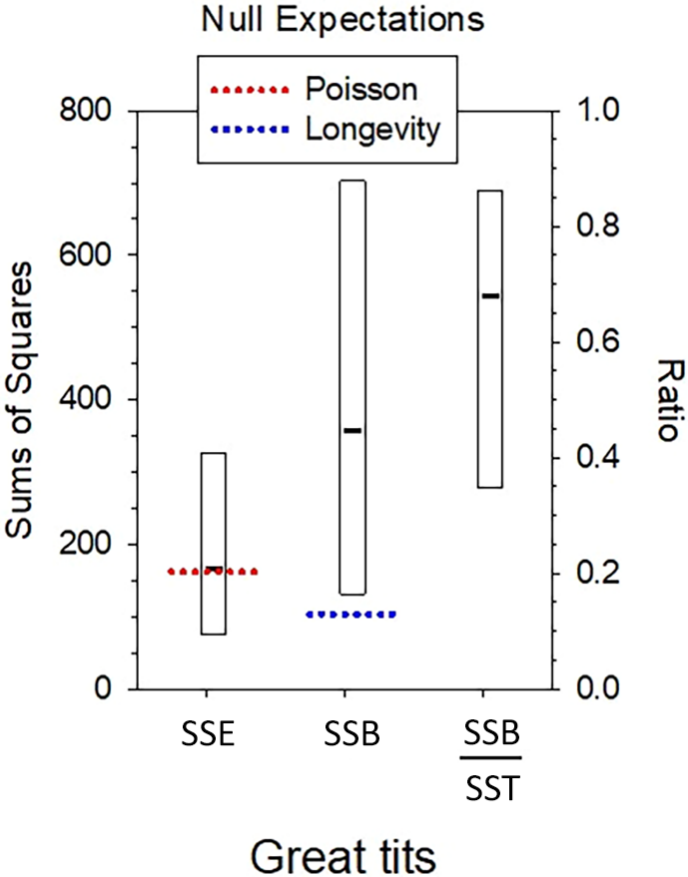
Results from bootstrapping raw data for lifetime reproduction in great tits. Black bars are point estimates discussed in the text; rectangles are empirical 95% confidence intervals across 10,000 bootstrap replicates. Left axis shows unbiased estimates of sums of squares; right axis shows SSB• as a fraction of the total sum of squares SST•. Dotted lines show the expected random contribution to SSE• from Poisson variance in reproductive success within ages and the expected contribution to SSB• from random variation in longevity.

LRS data can be more complicated to interpret because of potential correlations between reproduction and survival that can affect SSE•. In the great tit example, $\hat{\mathrm{SSE}•}$ was only slightly higher than (and statistically consistent with) a null model in which within‐age reproductive variance was Poisson and expected values of these correlations were 0 (Figure 5). Furthermore, the index of correlation between initial and subsequent reproductive success (*ρ*_*α,α+*_) was not significantly different from zero. It seems likely, therefore, that factors such as persistent individual differences and effects of reproduction on survival were not substantial, at least for this particular cohort.

The pie charts in Figure 4 are a convenient way to visually depict the partitioning of variance in reproductive success. It is immediately apparent, for example, that whereas within‐age effects dominate the overall variance in black bears, they are of relatively minor importance for great tits. Two of the most common practical applications for these results are calculations of effective population size and the Opportunity for Selection. In applied conservation and management, insights into factors that reduce the effective size: census size ratio can help direct efforts to raise that key ratio. For both male and female black bears, the most effective strategies would involve reducing disparities in reproductive success among individuals of the same age. For great tits, in contrast, individual variation in longevity and modest differences in fecundity with age are the primary factors that increase variance in LRS and reduce *N*_*e*_.

Ideally, *N*_*e*_ should be computed across a full life cycle; if not, the value applies to only part of a generation. One option is to compute variance in offspring number in terms of production of zygotes by zygotes (Hill, 1972), as doing so avoids the well‐known problem of creating an index that reflects both fertility of parents and survival of their offspring (Thomson & Hadfield, 2017). However, this predictably leads to zero‐inflation of ${\sigma_{k}^{2}}_{•}$ in proportion to the magnitude of mortality before first breeding (Waples & Reed, 2023). In the great tit example, ${\sigma_{k}^{2}}_{•}$ was estimated as production of age‐1 recruits by adults, which covered a full life cycle because maturity occurs at age 1. It is true that the Opportunity for Selection based on ${\sigma_{k}^{2}}_{•}$ computed this way will include components for both parental fertility (*I*•_*f*_) and offspring mortality (*I*•_*m*_), but these components would need to be studied separately in any detailed study of selection.

It should be remembered that the Opportunity for Selection is just that—an opportunity—and is not a demonstration that selection has actually occurred (see recent review by Reed et al., 2023). Nevertheless, the relative magnitude of OFS can help researchers direct scarce resources toward experimental designs that are most likely to produce interesting results. The indices used here ($\Delta_{I}$ for annual reproduction in black bears and $\Delta_{I•}$ for LRS in great tits) are independent of mean fitness and reflect the component of OFS that exceeds the random expectation under common null models. From Figure 4, it is apparent that OFS is relatively large for male black bears, and that within‐age effects would be the most productive factors to explore. Inspection of Table 3 indicates that ages 6 and older (with high variance‐to‐mean ratios) are particularly good candidates for studies of natural selection. For this particular cohort of great tits, $\Delta_{I•}$ is relatively small (Figure 4). It would be premature to conclude that selection is unimportant in this species, however, because this estimate applies to a single cohort of females, none of which survived beyond age 4. Furthermore, longitudinal studies of great tits demonstrate ample genetic variation for traits such as mean laying date, which can vary across years with different environmental conditions (Visser et al., 2021).

Because the equal‐variances ANOVA assumption will often be violated with empirical reproductive‐success data, Fisher's *F* statistic is not recommended in significance testing of variance partitioning. Instead, bootstrapping can be used with the raw data, as in the LRS example for great tits. The worked example for annual reproduction in black bears used age‐specific vital rates rather than raw data, because vital rates are widely available in standard life tables, but a similar bootstrap approach could be used to generate confidence intervals when raw data are available. Variance partitioning for annual reproduction does require an age‐specific vital rate ($\phi_{x}=\sigma_{k,x}^{2}/b_{x}$) that is not widely reported. If estimates of $\phi_{x}$ are not available, a range of plausible values could be explored.

Extrapolating from annual vital rates to LRS requires some assumptions about temporal correlations in individual reproductive success over time, which might be feasible for some species. For example, consequences of skip breeding might be modeled using the parameter *θ*_*i*_ (Shaw & Levin, 2013), which gives the probability that an individual will reproduce in the current year, given that it last reproduced *i* years previously. Persistent individual differences can be modeled using *theweight* algorithm (Waples, 2022), which could be tuned to simulate a desired level of positive correlation in individual reproductive success over time.

## AUTHOR CONTRIBUTIONS

**Robin S. Waples:** Conceptualization (equal); data curation (equal); formal analysis (equal); methodology (equal); software (equal); writing – original draft (equal); writing – review and editing (equal).

## CONFLICT OF INTEREST STATEMENT

The author declares no conflict of interest.

## Supporting information


Appendix S1

## Data Availability

Most of the results presented here are from simulations, R code for which is available in Appendix S1. Vital rates used in the black bear example were published in Waples et al. (2018). Data used in the great tit example were provided by Marcel Visser, The Netherlands Institute of Ecology, Wageningen, The Netherlands, and are available in Supporting Information. R code that reads generic input files and conducts the kinds of analyses illustrated in the worked examples (PartitionAnnual.R, which reads a file of age‐specific vital rates; and PartitionLifetime.R, which reads raw lifetime reproductive success data) is available on Zenodo (https://doi.org/10.5281/zenodo.10019554).

## Endnote

^1^
This is smaller than that shown in Waples et al. (2018) because the latter used modeled age‐specific variances smaller than those computed directly from the raw data in this example.
