# Supplementary material for: Partitioning variance in reproductive success, within years and across lifetimes
Source: Ecol Evol. 2023 Nov 20;13(11):e10647. doi: 10.1002/ece3.10647 (PMC10660325; doi:10.1002/ece3.10647)
Supplement: Supplementary file 1 — Appendix S1 [file ECE3-13-e10647-s001.zip › ece310647-sup-0002-SupplementaryS2.docx]

**Information regarding R code**

**PartitionAnnual.R**

This code partitions annual variance in offspring number into within-age and between-age components, and does a similar partitioning for the ratio Nb/N, where N is the number of adults alive in a given year and Nb is the effective number of breeders, and for the Opportunity for Selection. The program reads in a file named Vitals.csv, which contains a matrix of age-specific vital rates: age (indexed as x, starting with age 1), sx (probability of surviving from age x to x+1), bx (expected number of offspring that survive to age 1 produced by an individual at age x), and vk (variance in offspring number associated with each bx value). The first 3 variables are found in standard life tables, but the age-specific variances are seldom reported. Setting vk = bx produces random Poisson variance among individuals of the same age and sex. The code analyzes data for a single sex, so separate input files should be used for males and females.

In addition to supplying the input file, the user:

1. Specifies the number of age-1 offspring produced each year by each sex (N1); this allows the program to formally account for uneven primary sex ratio
2. Whether the last age class is a ‘plus’ age class containing all older individuals; if so, the number in the plus age class must be specified;
3. Whether the vector of numbers of individuals at age (Nx) is rounded to nearest integer or not.

The function ‘demography’ computes the cumulative survivorship function (Lx) and rescales the bx and vk vectors to values expected in a population of constant size, if that is not already the case.

The program then proceeds to calculate SSE and SSB statistics per a standard ANOVA analysis, using the rescaled bx and vk vectors. Because it is assumed that the age-specific variances in the input file are unbiased sample estimates, overall SSE is the unbiased estimate of the within-age component of overall variance. In contrast, overall SSB includes a contribution of random sampling error in estimating age-specific fecundity from samples, so the unbiased estimate (SSBhat) is obtained by subtracting the expected amount of the random component, which is a function of the scaled vk values. These various calculations are summarized in the output matrix, Partition. The first pie diagram partitions the overall sums of squared deviations in offspring number (SST = SSE+SSB) into random and greater-than-random components of both SSE and SSB.

The program then calculates Nb and Nb/N, where N is the number of adults (all ages with bx>0). The key parameters for Nb are the overall mean (Annualkbar) and variance (AnnualVk) in offspring number among all adults in a given year. AnnualVk is easily calculated using the definition of a variance as E(x^2^) – [E(x)]^2^. A second pie diagram is produced, as follows. If all adults have the same expected fecundity and reproductive success is random, the whole population behaves as a single Wright-Fisher ‘ideal’ population, with Nb/N = 1. The second pie diagram, which has an area of 1 unit, therefore depicts the degree to which greater-than-random within-age and between-age variances reduce Nb/N. The area of the black segment represents the Nb/N ratio, the area of the blue segment reflects reductions to Nb/N due to within-age effects, and the area of the red segment reflects reductions to Nb/N due to between-age effects.

The sample input file replicates the analysis of male black bears discussed in the text.

**PartitionAnnual.R**

This code partitions lifetime reproductive success into within-age, between-age, and longevity components. The program reads in a file named LRSdata.csv, the supplied version of which contains the great tit data. The dataset should represent a single birth cohort of individuals, and each line gives the number of offspring produced by one individual in one year, along with its age and unique ID. Parental ages are coded with respect to the ages in the adult lifespan, so (for example), if age at maturity is 3, a parent’s reproductive output for age 3 is recorded as age 1. The program computes raw (sample) sums of squares, then rescales them to expectations under constant N for the final analysis. The program reports results for the input data, then bootstraps the raw data to generate empirical CIs around the variance partitioning.
